# Supplementary figures and images for: Horizontal transfer of ICEclc-like elements in Pseudomonas aeruginosa clinical isolates
Source: J Bacteriol. 2026 Jun 12;208(7):e00009-26. doi: 10.1128/jb.00009-26 (PMC13393423; doi:10.1128/jb.00009-26)

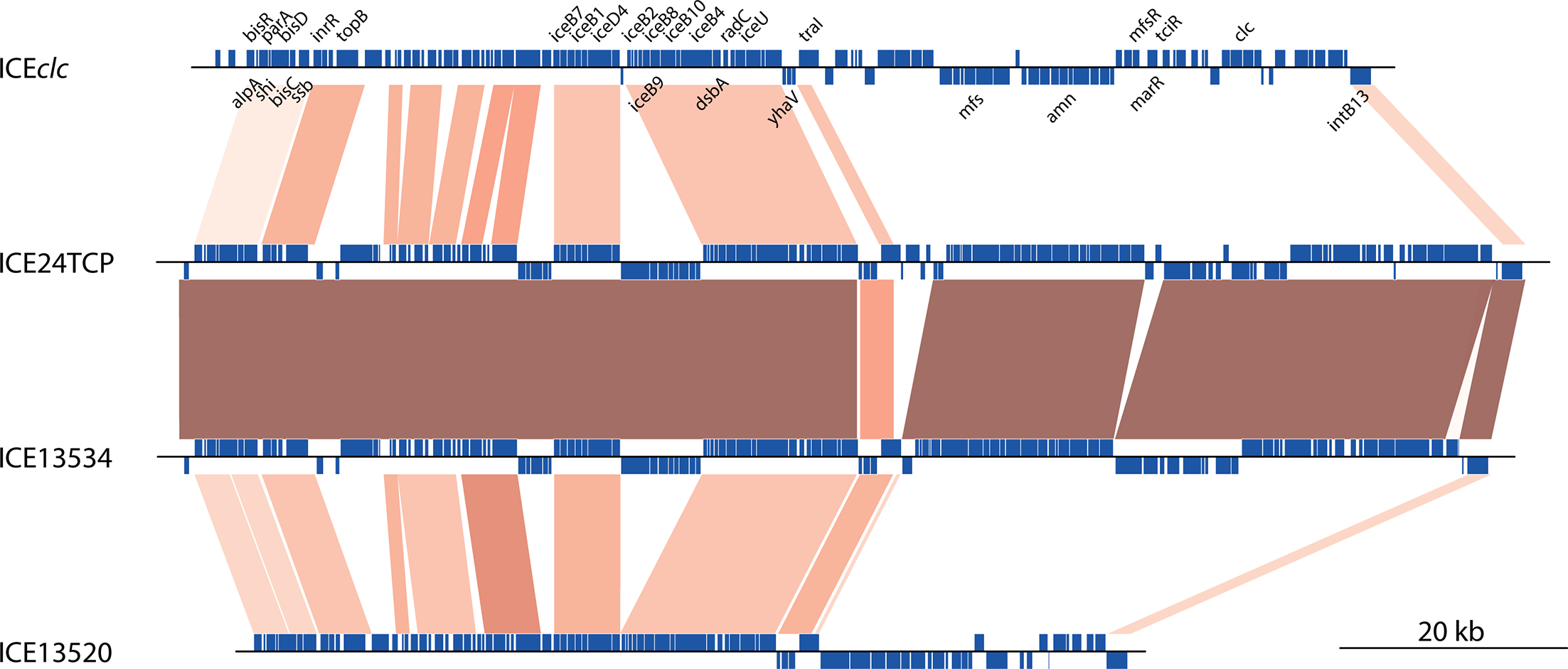

Supplement: Fig. S1 — Gene conservation and synteny comparisons between ICEclc and three P. aeruginosa ICEclc-family elements. Predicted ORFs and their orientation are represented by blue boxes on top (forward) or bottom (reverse strand). Red shades in between ICEs indicate regions of significant BLASTN homology (hits above default BLASTN thresholds, according to color-scale, with darker colors indicating higher homology). General conservation of a ‘core’ region is clearly visible, as well as individual cargo gene content. Known genes of ICEclc are indicated (simplified per operon, e.g., clc for the clcRABDE operon). [file jb.00009-26-s0001.tif]

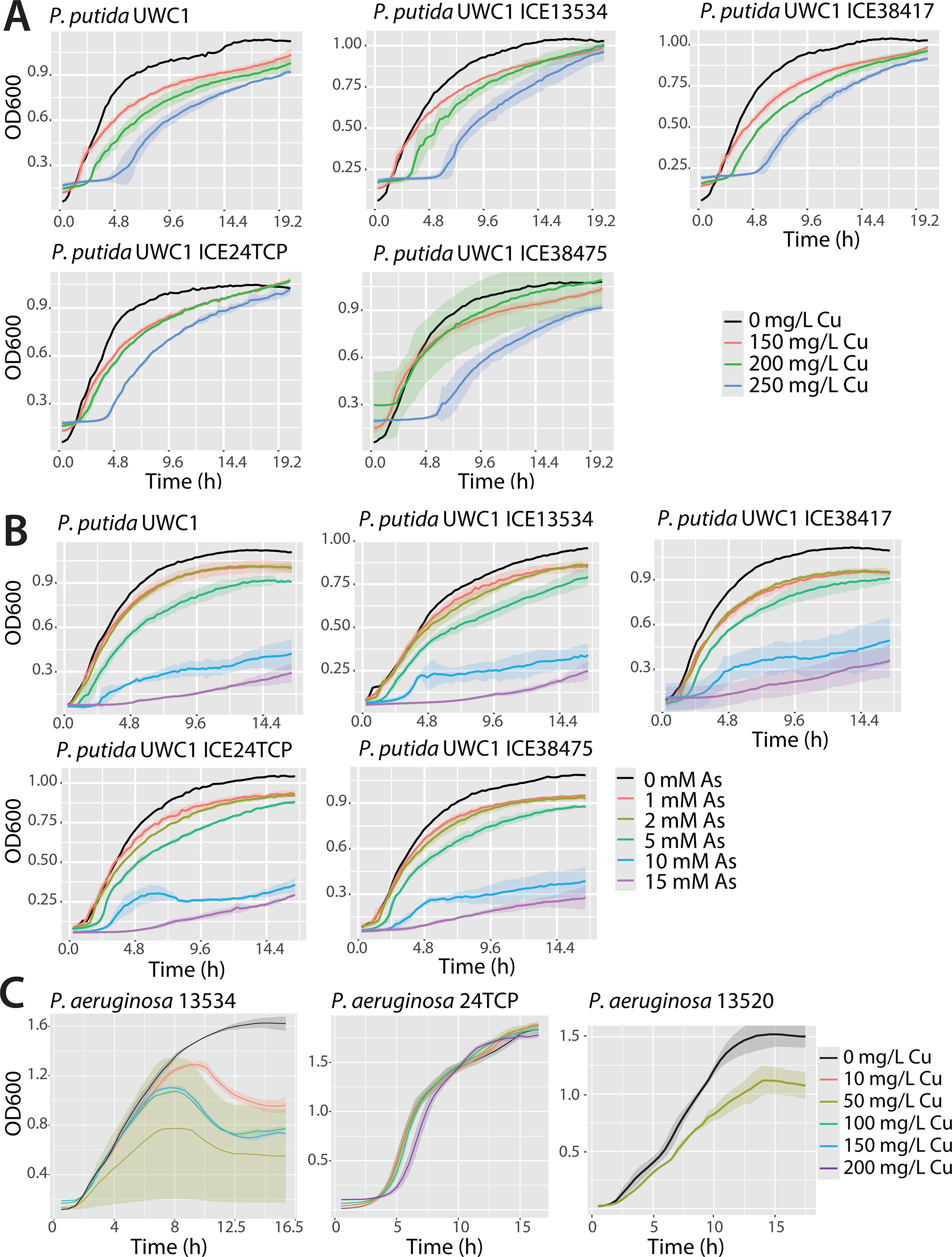

Supplement: Fig. S2 — (A-B) Growth curves of P. putida parent strain and four transconjugants carrying distinct P. aeruginosa ICEs in LB medium with increasing concentrations of copper and arsenite. (C) Growth curves of P. aeruginosa 13534, 24TCP, 13520 in LB with or without addition of copper at the indicated concentrations. [file jb.00009-26-s0002.tif]
